# Encoding Information From Rotations Too Rapid To Be Consciously Perceived as Rotating: A Replication of the Motion Bridging Effect on a Liquid Crystal Display

**DOI:** 10.1177/2041669520925111

**Published:** 2020-05-25

**Authors:** Maximilian Stein, Robert Fendrich, Uwe Mattler

**Affiliations:** Department of Experimental Psychology, University of Goettingen; Department of Psychological and Brain Sciences, Dartmouth College, Hanover, NH, USA; Department of Experimental Psychology, University of Goettingen

**Keywords:** motion aftereffects, motion perception, unconscious perception, liquid crystal display, temporal frequency

## Abstract

A ring of points that is rotated so rapidly is perceived as a stationary outline circle that can induce an illusory rotation with the same spin direction in a subsequently presented ring of stationary points. This motion bridging effect (MBE) demonstrates that motion information can be conveyed by temporal frequencies generally thought to exceed the processing capabilities of the human visual system. It was first described in displays shown with an analog oscilloscope, but the rapid rotation rates needed to produce the MBE have heretofore prevented it from being investigated with conventional raster scan monitors. Here, we demonstrate the MBE can be reliably generated using the new generation of 240 Hz LCD gaming monitors, and exhibits basic characteristics similar to those reported previously. These monitors therefore provide a readily available resource for research on the MBE and the studies of the visual processing rapid motions in general.

The conscious perception of spatial or temporal changes in the environment is limited by the visual system’s ability to perceptually resolve high spatial and temporal frequencies. This limit is approximately 60 cycles per degree of visual angle when discriminating the orientation of fine grids (He & MacLeod, 1996), 30 to 60 Hz when observing flickering patches (Cornsweet, 1970; Hartmann et al., 1979; Kelly, 1961), and approximately 30 Hz when detecting the direction of sinusoidally moving gratings (Burr & Ross, 1982). These limits, however, apply to conscious perception. Some studies suggest that information can unconsciously be encoded even when these limits are exceeded. He and MacLeod (2001), for example, demonstrated that gratings indistinguishable from a uniform field can affect the magnitude of the tilt aftereffect, and Shady et al. (2004) showed that temporal frequency modulations above the critical flicker fusion frequency (CFF) can influence the sensitivity to subsequent flickering patches.

In 2010, Mattler and Fendrich published a study that indicated the human visual system can process motions so rapid they are not visually perceptible as motion. On an analog oscilloscope with an effective frame rate of 1000 Hz, they presented a 16-point ring (the *inducing ring*^1^) that rotated at angular velocities of up to 2,250°/s. These high velocities were achieved by advancing the points around the ring circumference with repetition rates of up to 100 Hz at every display location. As the velocity and refresh rate of point positions along the ring circumference was increased, participants’ ability to judge the rotation direction of the ring drastically decreased, and the rotating points were seen as forming a static circular outline. However, when a stationary ring of 16 points (the *test ring*) preceded or followed the rotating ring, participants perceived an illusory motion that was predominantly in the direction of the inducing ring. When participants were asked to describe the source of the motion precept in a small set of supplementary trials (Phenomenological Test Procedure 4), it was attributed solely to the test ring 58% of the time and to both rings 31% of the time. Mattler and Fendrich argued that this effect demonstrated that the rapid advance of the rotating points, though not consciously perceived by participants, conveyed a directional motion signal to the test ring points.

These findings were extended by Stein et al. (2019), who showed how the motion bridging effect (MBE), indexed by the congruence of the inducing and test ring directions, was affected by variations in the diameter of the rings, the number of the points used to form those rings, and the spacing of the points along the ring perimeters. Stein et al. proposed that the perceived rotation of the test ring might be related to apparent motion. According to this view, the MBE would reflect the joint action of two processes, one that registers the direction of the inducing ring’s rotation and one that generates the illusory test ring spin.

Previous MBE studies have been conducted using analog oscilloscope displays with the point positions on the cathode ray tube (CRT) screen controlled by Digital-to-Analog converters and screens customized with a fast phosphor. Conventional computer monitors with screen refresh rates below 100 Hz, while widely employed for investigations of phenomena like visual attention, are unsuitable for displaying the controlled rapid ring rotations needed for MBE investigations. However, recent technical advances have made liquid crystal display (LCD) *gaming monitors* with refresh rates of up to 240 Hz readily available. These high refresh rate monitors allow display sequences to be updated more rapidly than has been possible with the standard CRT monitors. Moreover, LCD monitors avoid potential artifacts associated with the downward raster scans CRT monitors use to display each frame: All pixels are effectively replaced simultaneously. With CRT monitors, phosphor decay times impose an additional limitation on the speed with which displays can be updated. A corresponding limitation in LCD monitors is imposed by LCD switching times. Although in early LCD monitors these switching times were too slow to allow the use of these monitors in psychophysical research, technological advances have now overcome this problem (Wang & Nikolić, 2011; Zhang et al., 2018).

We wondered whether currently available LCD gaming monitors with 240 Hz would have sufficient speed and spatial resolution to be used to study the MBE. Pilot observations suggested that this was in fact possible. The experiments reported here formally confirm these observations. We replicate the MBE and certain of its characteristics with this technology and compare the MBE on an LCD monitor to the MBE on an oscilloscope CRT.

## Method

### Participants

The participants were 12 students at the University of Göttingen with a mean age of 22.5 years. They were compensated €7 per hour for their participation or received student credits. Testing conducted with the Landolt ring chart confirmed all participants had normal or corrected-to-normal vision. After providing their written informed consent, participants completed one session that took approximately 1 hour.

### Apparatus

Stimuli were presented on a 240 Hz LCD monitor (Dell Alienware AW2518HF) controlled by a PC via the display port connector of a MSI GeForce GT 1030 graphics card. The experiment was run in a darkened room and participants had their head positions stabilized by a chin and forehead rest 108 cm from the LCD monitor. This resulted in a per-pixel display size of 0.015° of visual angle. The background luminance of the LCD was reduced to a minimum (0.02 cd/m^2^).

**Figure 1.**
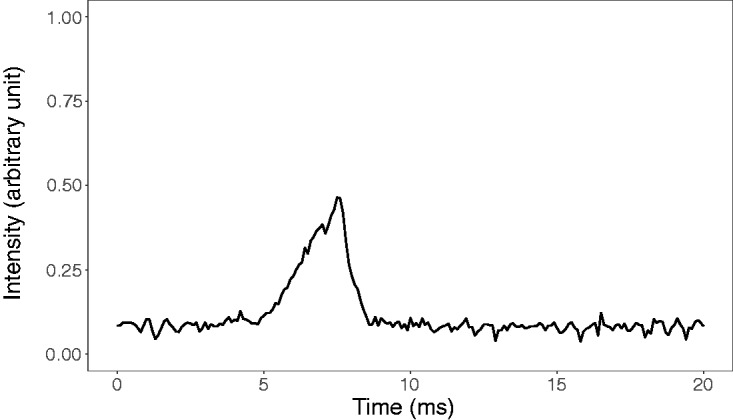
The Time Course of Two Luminance Changes From 0.02 cd/m^2^ to 1.5 cd/m^2^ and Back to 0.02 cd/m^2^ Across Two Frames on the LCD screen. These luminance shifts correspond to that what happened during the presentation of a single inducer point in the experiment. The measurement interval is 20 ms with the frame switches occurring at approximately 3.3 and 7.5 ms.

To find out if the pixels of the monitor update fast enough to keep up with its refresh rate, we showed a white area on the screen for one frame and recorded the light intensity on a point of the screen with an optical transient recorder (Display-Metrology & Systems OTR-3). Figure 1 shows that the light intensity gradually increases and then returns to its initial level during a period of time that is shorter than the 4.167 ms frame duration. In consequence, the stimuli presented in each screen do not interact with the stimuli in the following screen in a manner that could indicate the direction of rotation.

### Stimuli

**Figure 2.**
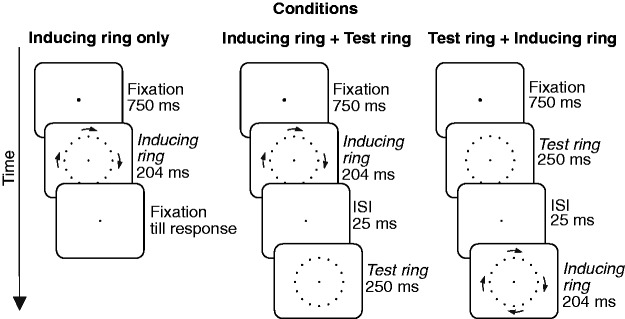
Display Sequence in the Three Conditions of the Experiment. On a given trial, the inducing ring rotated either clockwise (as shown in the figure) or counterclockwise. In the inducing-ring-only condition, there was no test ring present while in the other two conditions the test ring either preceded or followed the inducing ring. The dots on the LCD appeared light gray on a dark gray background.

Stimuli are illustrated in Figure 2. Participants were presented with a rapidly rotating inducing ring and a stationary test ring constructed from 16 equally spaced dots. The diameter of both rings was 6° and the diameter of each dot was 0.12° of visual angle. The luminance of the test ring dots, measured with a Minolta LS-100 luminance meter with a close-up lens, was 3.0 cd/m^2^ and the luminance of the inducing ring dots was 1.5 cd/m^2^. Note that when running the monitor in 240 Hz mode, the luminance does not reach its maximum when only one frame is displayed. Since this was the case for the points that formed our inducing rings, we determined their luminance by presenting a flickering square with a one-frame (4.167 ms) on-off duty cycle and doubled the measured luminance of this square.

At the monitor’s 240 Hz refresh rate, frames were updated every 4.167 ms. The inducing ring was rotated at angular velocities of 225, 675, 900, and 1,350°/s by, respectively, advancing all of its dots by 0.9375, 2.8125, 3.75, and 5.625 angular degrees per frame in a clockwise or counterclockwise direction. These distances respectively generated 384, 128, 96, and 64 dot positions along the ring circumference, which were refreshed at respective temporal frequencies of 10, 30, 40, and 60 Hz as successive dots crossed each display location. The rotation of the inducing ring always started and ended with dots placed at the same set of ring circumference positions. These were also the positions of the test ring dots when the test ring was presented. At 10 Hz temporal frequency, the inducing ring looked like a rotating outline circle formed by overlapping dots. At higher temporal frequencies, it appeared to be a circle of stationary dots, almost touching at 30 Hz but clearly separated at 40 Hz and more widely separated at 60 Hz.

### Task

Participants reported the direction of any perceived rotation (clockwise or counterclockwise). Responses were registered with the right and left arrow keys of a conventional computer keyboard, to indicate clockwise rotation and counterclockwise rotation, respectively. Trial blocks were started by a press of the space key. No feedback was given about the correctness of responses.

### Procedure

Participants were instructed to maintain their gaze on a central ﬁxation point during the trials. This fixation point brightened for 750 ms at the start of each trial to indicate that the inducing ring or test ring was about to appear. In the conditions in which the test ring was presented it followed or preceded a 204 ms inducing ring presentation with a 25 ms interstimulus interval and remained visible for 250 ms. Subject’s reports of the perceived motion direction were recorded starting 300 ms after the offset of the last presented ring. A response was required for the experiment to proceed. A new trial started 1 second after the response.

### Design

Fifteen trial blocks were run in each session with the first three blocks treated as practice and excluded from data analysis. There were 48 trials in each block. Blocks were run in a repeated sequence: A block with the inducing ring only was followed by a block with the inducing ring preceding the test ring, and a block with the test ring preceded the inducing ring. The temporal frequency and the rotation direction of the inducing ring were varied quasi-randomly within each block.

The combination of three *Test Ring* conditions (absent, preceding, and following the inducing ring) and four *Angular Velocities* of the inducing ring (225, 675, 900, and 1,350°/s) produced 12 experimental conditions. There were 48 trials in each of these conditions: 24 with a clockwise inducing ring rotation and 24 with a counterclockwise rotation.

### Statistical Analysis

We used signal detection methods to analyze performance (Macmillan & Creelman, 2005). We defined hits as clockwise responses to a clockwise rotation and false alarms as clockwise responses to a counterclockwise rotation. Values of *d* ′ were estimated by measuring hit and false alarm rates separately for each subject in each condition and correcting these values by applying the log-linear rule (Hautus, 1995). Across participants, mean *d* ′ measures were analyzed with a two-way repeated measures analysis of variance (ANOVA) which evaluated the effect of angular velocity and test ring condition on observers’ sensitivity to the inducing ring rotation. We will refer to these factors as *Angular Velocity* and *Test Ring*. All reported ANOVA *p*-values were corrected using Greenhouse-Geisser estimates of sphericity, but for the sake of readability, the uncorrected degrees of freedom are reported. Differences between specific conditions were evaluated with post hoc Bonferroni corrected two-tailed *t* tests. Performance levels in specific conditions were compared to a chance level of 50% with one-tailed Bonferroni corrected *t* tests that evaluated whether *d* ′ exceeded zero.

## Results

**Figure 3.**
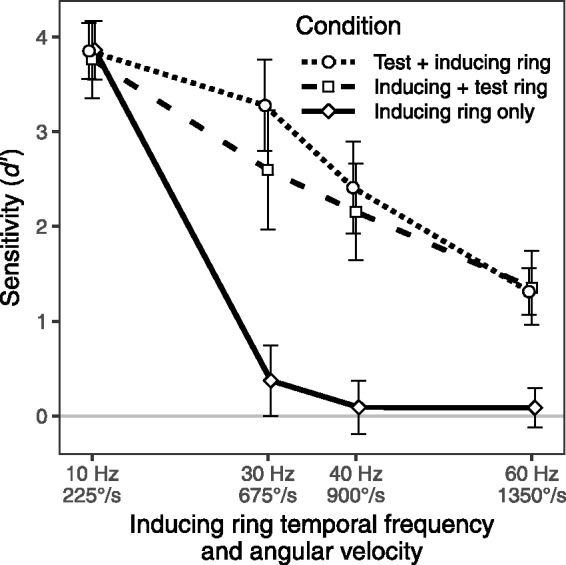
Mean sensitivity (*d* ′) as a function of the angular velocity of the inducing ring in the inducing ring only condition (the solid black line), the condition where the inducing ring precedes the test ring (the dashed line), and the condition where the test ring precedes the inducing ring (the dotted line). The solid gray line indicates the chance level of accuracy (*d* ′ = 0). The error bars show 95% confidence intervals. Points and confidence intervals are slightly offset horizontally to improve their visibility.

Means and confidence intervals for sensitivity to the inducing ring rotation direction in the 24 conditions are shown in Figure 3. These data are also presented in terms of mean percent correct accuracy rates in Table 1. ANOVA outcomes are presented in Table 2. The main effects of both *Angular Velocity* and *Test Ring* were significant and there was a significant *Angular Velocity* × *Test Ring* interaction. Critically, mean sensitivity differed conspicuously in the three *Test Ring* conditions (see Figure 3). When the inducing ring was presented alone, mean sensitivity was high in the 10 Hz condition, but declined steeply to zero as *Angular Velocity* was increased (*d* ′ = 3.86, 0.37, 0.09, and 0.09 with 225, 675, 900, and 1,350°/s, respectively). When the inducing ring was followed or preceded by the test ring, mean sensitivity declined linearly and stayed above zero even at the highest Angular Velocity employed. When the inducer preceded the test ring, *d* ′ values of 3.77, 2.60, 2.15, and 1.35 were observed with 225, 675, 900, and 1,350°/s, respectively. When the test ring preceded the inducer, *d* ′ values of 3.85, 3.28, 2.41, and 1.31 were observed with these velocities. The contrast between the rapid decline to chance sensitivity in the inducer only condition and the gradual decline in conditions with the test ring is the source of the interaction *Angular Velocity* × *Test Ring* and reflects a basic characteristic of the MBE.

**Table 1.** Mean Percentage of Correct Direction Reports.

	Angular velocity (in °/s)			
Condition	225	675	900	1,350
Inducing ring only	98.6	56.3	51.2	51.4
Inducing ring + Test ring	98.1	88.2	84.0	71.9
Test ring + Inducing ring	98.8	95.0	87.5	73.3

**Table 2.** Outcomes of Analysis of Variance (ANOVA).

Effect	Numerator *df*/Denominator *df*	*F*	*p*
*Angular Velocity*	3/33	215.58	<.001
*Test Ring*	2/22	72.35	<.001
*Angular Velocity* × *Test Ring*	6/66	30.54	<.001

*Note.* The ANOVA was calculated on the *d* ′ values.

**Figure 4.**
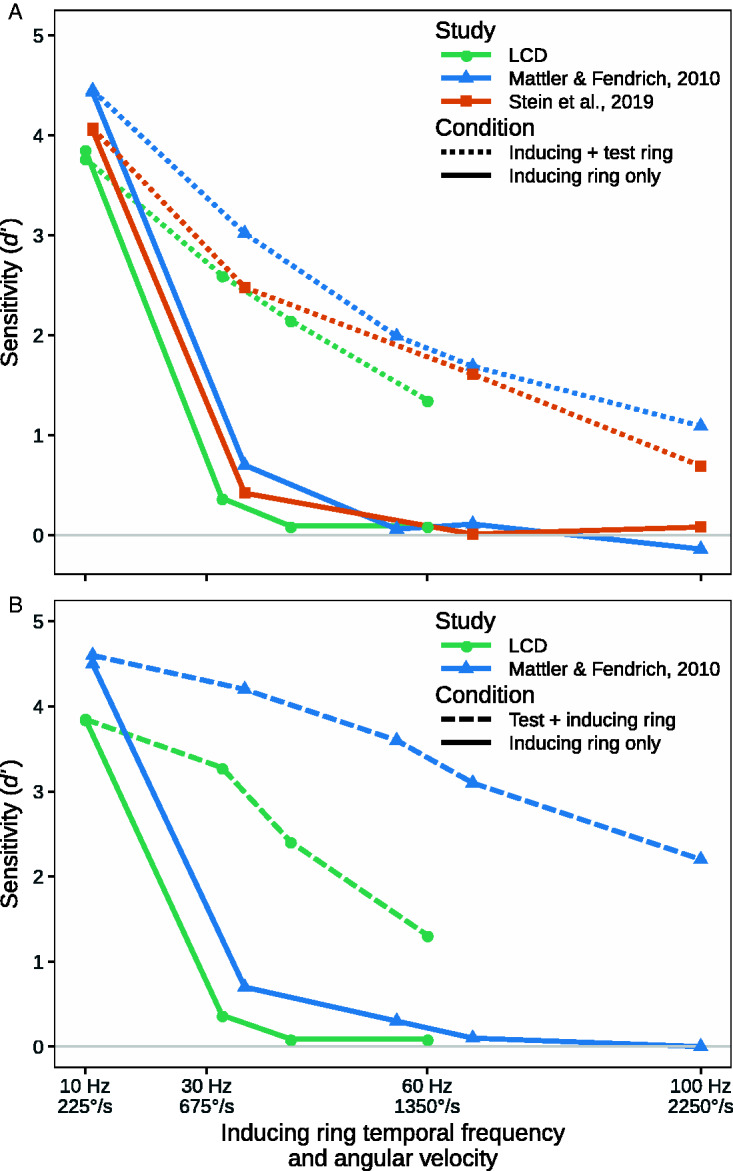
Mean sensitivity (*d* ′) as a function of inducing ring angular velocity in the inducing ring only conditions (the solid black line) and the conditions where the test ring was presented (the dashed lines) in this study and its predecessors (Mattler & Fendrich, 2010; Stein et al., 2019). Panel A presents data from conditions where the test ring followed the inducing ring and Panel B where it preceded the inducing ring. In all studies, the mean sensitivity with a 16-point inducing ring is reported. The solid gray line indicates the chance level of accuracy (*d* ′ = 0).

Importantly, the improvement in sensitivity to the inducing ring direction produced by the test ring presentation occurs even when subjects perform at chance in the inducing ring only condition. In the present investigation, four 1-tailed *t*-tests were run (one for each inducer velocity in the inducing ring only condition) to compare participants sensitivity to the inducing ring direction to a chance level of 50% (*d* ′ = 0). When a Bonferroni adjusted alpha level of .0125 was employed, the mean *d* ′ in the inducing ring only condition was significantly greater than zero only when the Angular Velocity was 225°/s (*p* < .001) and did not exceed zero in conditions with higher velocities (*p =* .025, *p =* .248, and *p =* .194, for 675, 900, and 1,350°/s respectively). However, sensitivity was slightly better than chance with 675°/s when an uncorrected alpha level of .05 was employed, suggesting with this velocity it was sometimes possible for participants to detect the rotation direction. As can be seen in Figure 4, the pattern of results is very similar to that observed in the previous studies, which used oscilloscope displays (Mattler & Fendrich, 2010; Stein et al., 2019), and is consistent with Burr and Ross’s study (1982), in which the detection of the motion of sinusoidal gratings starts to break down at around 30 Hz, which corresponds to an Angular Velocity of 675°/s.

While the contrast between the inducing-ring-only and the two test-ring-present conditions is the most striking aspect of Figures 3 and 4, it can also be seen that the two test-ring-present conditions are not identical. Previously, Mattler and Fendrich (2010) reported that the MBE is larger when the test ring precedes the inducing ring than when it follows the inducing ring with velocities of 750°/s and higher. One-tailed post-hoc *t*-tests (with a Bonferroni adjusted alpha level of .0167) confirmed that a similar difference is also present in the current data when the Angular Velocity is 675°/s (*p* < .005), but not in the 900 and 1,350°/s conditions. In addition, when the test ring precedes the inducing ring, participants sensitivity falls short in the present data when compared to previous data (Figure 4B). Both, characteristics of the LCD screen and changes to the stimulus parameters used may have contributed to these differences.

## Discussion

The ability of observers to perceive the motion of repetitive patterns is limited by the temporal frequency of the intensity modulations that those motions generate (Burr & Ross, 1982). The rapid rotations of the ring stimuli needed to demonstrate the MBE produce modulations too rapid to support conscious motion percepts, but not so rapid they prevent the encoding of information that can reveal the direction of the rotations. To study the processing of rapid motions of this kind requires a technology that enables screen displays to be updated at very rapid rates. This has previously been achieved by using analog oscilloscopes as the display device. Here, we demonstrate that recent LCD monitors with 240 Hz frame rates can also be used to investigate these rapid motions.

In the present study, participants could report the direction of an inducing ring rotation when the dots that formed the ring perimeter advanced at angular velocities of 225°/s and were refreshed at 10 Hz, but their performance decreased to near chance when the velocity was 675°/s (refresh rate 30 Hz), and completely to chance at velocities of 900°/s (refresh rate 40 Hz), and 1,350°/s (refresh rate 60 Hz). The appearance of the inducing ring accords with this outcome: At refresh rates of 30 Hz and higher, it is seen as a static circle of flicker-free dots. This ring of dots corresponds to the steady outline circle that was seen at high inducing ring velocities in previous MBE experiments conducted with oscilloscope screens. However, despite the static appearance of the inducing ring dots, when the inducing ring is followed by the veridically stationary 16-dot test ring, the test ring dots appear to briefly rotate, primarily in the same direction as the invisible inducing ring rotation. Similarly, when a stationary test ring precedes the inducing ring, that test ring appears to momentarily spin in the inducing ring direction when the inducing ring onsets. Mattler and Fendrich (2010) used the frequency of the reports in which the illusory test ring rotation matched the actual (although not consciously visible) direction of the inducing ring rotation as a quantitative measure of the MBE. The congruency rates obtained in the present study with comparable inducing ring velocities are similar to the rates they obtained.

The fact that in the 675°/s condition performance is significantly better when the inducer precedes the test ring than when it follows it suggests a possible difference in the mechanisms that produce the MBE in the two sequences. However, the slightly above chance performance in the 675°/s inducer only condition suggests that subjects consciously perceived the direction of inducing ring rotation on some trials. This finding constrains the interpretation of the test-ring first advantage since unbiased estimates of the MBE require that the inducer rotates too fast for its direction to be consciously perceived. Also, as noted earlier, a more pervasive test-ring-first advantage was reported by Mattler and Fendrich (2010). The extent to which a difference between the oscilloscope and LCD displays contributes to this effect remains to be determined.

The MBE demonstrates the human visual system can derive motion information from stimuli with temporal frequencies previously thought to exceed its processing capabilities. With oscilloscope screens, this occurs with refresh rates of 100 Hz (Mattler & Fendrich, 2010) and higher (125 Hz in Stein et al., 2019). The present study shows that it is robust with an LCD screen when the inducing ring dots are refreshed at 60 Hz, which is at the upper limit of conventional CFF estimates (Kaufman, 1974) and exceeds the reported limits for the conscious detection of motion in repetitive stimuli (Burr & Ross, 1982; Kelly, 1979). The MBE therefore extends the gamut of phenomena that reveal the visual system’s processing of information that is not consciously visible. Note, however, that high-frequency information can also become visible when this information is transmitted via cortically implanted microelectrodes that produce light sensations called phosphenes (Brindley & Lewin, 1968).

The present study is the first to demonstrate that the MBE can be investigated with a readily available commercial system. It replicates the modulation of the MBE by the inducing ring’s angular velocity both when the test ring precedes and follows the inducing ring (Mattler & Fendrich, 2010; Stein et al., 2019). This replication with new software and hardware gives added credence to previous descriptions of the MBE. Moreover, the differences between the LCD and oscilloscope display provide a demonstration of the MBE’s generality. With the oscilloscope screen, the inducing and test rings were necessarily constructed from luminous points on a background close to totally black. On the LCD screen, however, slightly larger 0.12° dots were used and displayed on a dark gray background. In addition, the 240 Hz LCD frame rate remains well below the 1000 Hz effective frame rate used in the previous oscilloscope investigations. To achieve comparable inducing ring velocities with the lower frame rates, the dots had to be advanced in larger spatial steps. In consequence, the inducing ring looks like a chain of dots with the LCD rather than the continuous outline circle it appeared to be with the oscilloscope. Importantly, in both cases, the inducing ring conveys no motion percept when the temporal frequencies exceed 30 Hz. We note, however, that the absence of any visible motion in the inducing ring strikes us as especially salient with the LCD screen because of the conspicuously static character of the inducing ring dots. We also note that although the motion illusion looks very similar on the two devices, our informal impression is that it appears to be briefer with a more abrupt start and stop on the LCD screen than on the oscilloscope. The display attributes that might produce differences in the appearance of the illusion of this kind remains to be determined.

In conclusion, the present study suggests that LCD monitors are a viable tool for the investigation of the MBE. Used in conjunction with MRI or EEG, they may enable investigations of the physiological sources of the MBE. Although the frame rate of LCD monitors is still limited compared to that of the oscilloscope, they allow the effects of rapidly moving stimuli to be examined using variables that cannot be readily manipulated on an oscilloscope. These include manipulations of shape, complexity, color, background luminance, contrast, and size. By uncovering new features of the MBE, such studies could facilitate the development of new or more extensive theoretical accounts of the illusion.

## Note

1 The term “inducing ring” was chosen to be consistent with previous reports. It indicates only that the apparently continuous outline circle is required to produce the illusory motion percept in the test ring, and is not intended to have implications regarding the character or mechanism of this illusion.

## References

[bibr1-2041669520925111] Brindley G. S. Lewin W. S. (1968). The sensations produced by electrical stimulation of the visual cortex. The Journal of Physiology, 196, 479–493. 10.1113/jphysiol.1968.sp008519 4871047 PMC1351724

[bibr3-2041669520925111] Burr D. C. Ross J. (1982). Contrast sensitivity at high velocities. Vision Research, 22, 479–484. 10.1016/0042-6989(82)90196-1 7112947

[bibr4-2041669520925111] Cornsweet T. N. (1970). Visual perception. Academic Press.

[bibr5-2041669520925111] Hartmann E. Lachenmayr B. Brettel H. (1979). The peripheral critical flicker frequency. Vision Research, 19, 1019–1023. 10.1016/0042-6989(79)90227-X 532115

[bibr6-2041669520925111] Hautus M. J. (1995). Corrections for extreme proportions and their biasing effects on estimated values of *d**’* Behavior Research Methods, Instruments, & Computers, 27, 46–51. 10.3758/BF03203619

[bibr7-2041669520925111] He S. MacLeod D. I. A. (1996). Local luminance nonlinearity and receptor aliasing in the detection of high-frequency gratings. Journal of the Optical Society of America A, 13, 1139–1151. 10.1364/JOSAA.13.001139 8926545

[bibr8-2041669520925111] He S. MacLeod D. I. A. (2001). Orientation-selective adaptation and tilt after-effect from invisible patterns. Nature, 411, 473–476. 10.1038/35078072 11373679

[bibr9-2041669520925111] Kaufman L. (1974). Sight and mind: An introduction to visual perception. Oxford University Press.

[bibr10-2041669520925111] Kelly D. H. (1961). Visual responses to time-dependent stimuli. I. Amplitude sensitivity measurements. Journal of the Optical Society of America, 51, 422–429. 10.1364/JOSA.51.000422 13752375

[bibr11-2041669520925111] Kelly D. H. (1979). Motion and vision. II. Stabilized spatio-temporal threshold surface. Journal of the Optical Society of America, 69, 1340–1349. 10.1364/JOSA.69.001340 521853

[bibr12-2041669520925111] Macmillan N. A. Creelman C. D. (2005). Detection theory: A user’s guide (2nd ed.). Lawrence Erlbaum Associates.

[bibr13-2041669520925111] Mattler U. Fendrich R. (2010). Consciousness mediated by neural transition states: How invisibly rapid motions can become visible. Consciousness and Cognition, 19, 172–185. 10.1016/j.concog.2009.12.015 20093045

[bibr15-2041669520925111] Shady S. MacLeod D. I. A. Fisher H. S. (2004). Adaptation from invisible flicker. Proceedings of the National Academy of Sciences of the United States of America, 101, 5170–5173. 10.1073/pnas.0303452101 15051882 PMC387392

[bibr16-2041669520925111] Stein M. Fendrich R. Mattler U. (2019). Stimulus dependencies of an illusory motion: Investigations of the motion bridging effect. Journal of Vision, 19, 1–23. 10.1167/19.5.13 31100129

[bibr17-2041669520925111] Wang P. Nikolić D. (2011). An LCD monitor with sufficiently precise timing for research in vision. Frontiers in Human Neuroscience, 5, 1–10. 10.3389/fnhum.2011.00085 21887142 PMC3157744

[bibr18-2041669520925111] Zhang G.-L. Li A.-S. Miao C.-G. He X. Zhang M. Zhang Y. (2018). A consumer-grade LCD monitor for precise visual stimulation. Behavior Research Methods, 50, 1496–1502. 10.3758/s13428-018-1018-7 29532446

